# The Southern Homœopathic Association

**Published:** 1887-02

**Authors:** 


### THE SOUTHERN HOMŒOPATHIC ASSOCIATION.

The third annual meeting of this Association was held in New Orleans, December 8th, 1886.

An address was delivered by the President, Dr. A. L. Monroe.

The Committee on Legislation reported that in North Carolina, Alabama, and Mississippi, where there are Boards of Medical Examiners of the old school, their arrangements, however fair in appearance, are, from the nature of things, intimidating to applicants of other schools. The result is that few of our school enter those States; therefore, a set of resolutions was passed protesting against the prevailing medical legislation.

A paper was read by Dr. Henry, of Montgomery, upon the methods in vogue of preparing high potencies. He took the ground that there is too much of the ethereal or visionary in some of these preparations, as presented by the patentees of the machinery by which they are made, for reasonable men. He urged the greatest care in selecting not only the remedy, but the potency for each particular patient. The paper did not meet with very hearty approval.

Other papers were read upon Diphtheria, Sanitary Science—a very interesting paper; Surgery of Gunshot Wounds of the Spine, Nephoraphy, Excision of the Mammary Gland for Sarcomatous Neoplasms, Lacerations of the Female Perineum,

Bowman Method of Coffee and Milk-feeding of Infants, and the Indiscriminate Use of Alcoholic Liquors and Opiates.

Officers elected were: President, Dr. Joseph Jones, of San Antonio, Texas; First Vice-President, Dr. Walter M. Dake, of Nashville, Tenn.; Second Vice-President, Dr. E. A. Murphy, of New Orleans; Recording Secretary, Dr. C. G. Fellows, of New Orleans; Corresponding Secretary, Dr. C. R. Mayer, of St. Martinsville, La.; Treasurer, Dr. J. G. Belden, of New Orleans.

